# Peripartum Cardiomyopathy: A Case Series Emphasizing the Significance of Left Ventricular Global Longitudinal Strain Imaging

**DOI:** 10.7759/cureus.51331

**Published:** 2023-12-30

**Authors:** Digvijay D Nalawade, Sridevi Chigulapalli, Ajitkumar K Jadhav, Susheel K Malani, Madhura A Gandhi

**Affiliations:** 1 Cardiology, Dr. D. Y. Patil Medical College, Hospital & Research Centre, Dr. D. Y. Patil Vidyapeeth, Pune, IND; 2 Central Research Facility, Dr. D. Y. Patil Medical College, Hospital & Research Centre, Dr. D. Y. Patil Vidyapeeth, Pune, IND

**Keywords:** peripartum cardiomyopathy, left ventricular dysfunction, cardiology research, global longitudinal strain, echocardiography, heart failure

## Abstract

Peripartum cardiomyopathy (PPCM) poses diagnostic and management challenges, while left ventricular global longitudinal strain (LV GLS) provides crucial insights into myocardial function. This case series explores the significance of LV GLS imaging in diagnosing and managing PPCM. Three distinct cases of varying severity highlight the significance of LV-GLS in assessing myocardial function during the peripartum period. Each case exhibited varying degrees of cardiac impairment, with LV GLS serving as a sensitive indicator of dysfunction. Treatment response, closely monitored through LV GLS changes, emphasizes the importance of this imaging technique in evaluating therapy efficacy. The discussion underscores deviations from traditional management approaches, emphasizing the need for personalized strategies in PPCM. Nonetheless, the study's limitations stress the need for broader research to validate these findings across diverse populations and settings, as well as long-term follow-up of the patients who had persistence of abnormal global longitudinal strain values despite recovery of left ventricular ejection fraction.

## Introduction

Peripartum cardiomyopathy (PPCM) stands as a rare (an incidence of 0.75 per 1000 live births) but serious condition characterized by the onset of left ventricular (LV) systolic dysfunction during the latter stages of pregnancy or five months following childbirth, without any other identifiable cause [1,2]. In the diagnostic landscape of PPCM, left ventricular global longitudinal strain (LV GLS) emerges as an invaluable and sensitive tool for assessing myocardial function. LV GLS provides an elusive perspective on myocardial deformation, offering insights that extend beyond traditional measures like left ventricular ejection fraction (LVEF) and allows for the early detection of subtle changes in LV performance, contributing to a comprehensive understanding of cardiac dynamics during the peripartum period [3]. This case series aims to underscore the significance of incorporating LV GLS assessment in the comprehensive evaluation of PPCM, emphasizing its potential to enhance early detection, risk stratification, and overall management of this challenging cardiac condition during the peripartum period.

## Case presentation

Case 1

**Figure 1:**
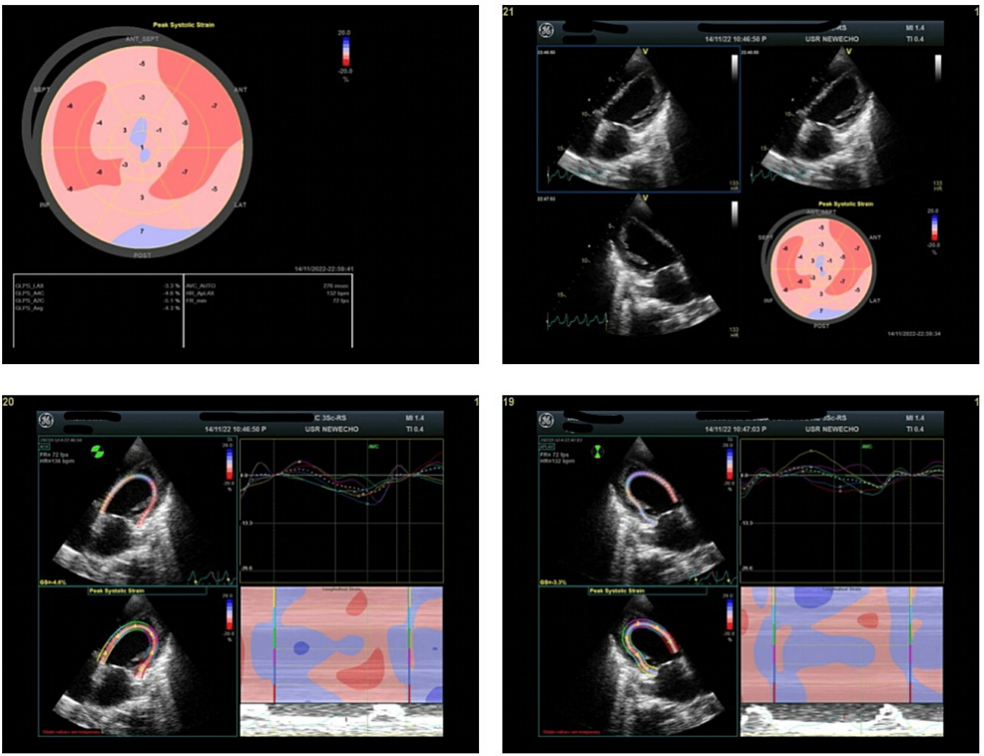
Case 1: Bull's eye image of left ventricular global longitudinal strain imaging Bull's eye image of left ventricular global longitudinal strain imaging showing significant reduction in average global longitudinal strain values.

A 31-year-old primigravida presented with acute decompensated heart failure at 34 weeks of gestation. Her history included conception three years after marriage, and she had undergone a lower segment cesarean section (LSCS) at 36 weeks. Her ECG showed atrial flutter with 2:1 conduction and echocardiography showed global LV hypokinesia with LVEF of 22% and LV GLS of -4.3% (Figure 1), indicating severe impairment of cardiac function. N-terminal pro-B-type natriuretic peptide (NT-proBNP) level was 7830 pg/ml on admission. She was a known case of hypothyroidism with no history of gestational diabetes or pregnancy-induced hypertension. She received ramipril for its cardioprotective effects. Carvedilol was administered to reduce cardiac workload and improve function. Torsemide was prescribed to manage fluid overload and reduce symptoms of heart failure. Amiodarone was administered to control tachyarrhythmia. Bromocriptine was not given, indicating a deviation from the traditional management approach that includes bromocriptine for its potential benefits in PPCM. Her response to treatment was closely monitored. Regular assessments of LV GLS were conducted to gauge the myocardial response to therapy. Adjustments to medications were made as needed based on clinical and imaging data. Over the subsequent weeks, the patient showed improvement in symptoms. LV GLS values demonstrated a positive response, indicating a recovery of myocardial function. Continuous monitoring ensured appropriate adjustments to her medication regimen.

Case 2

**Figure 2:**
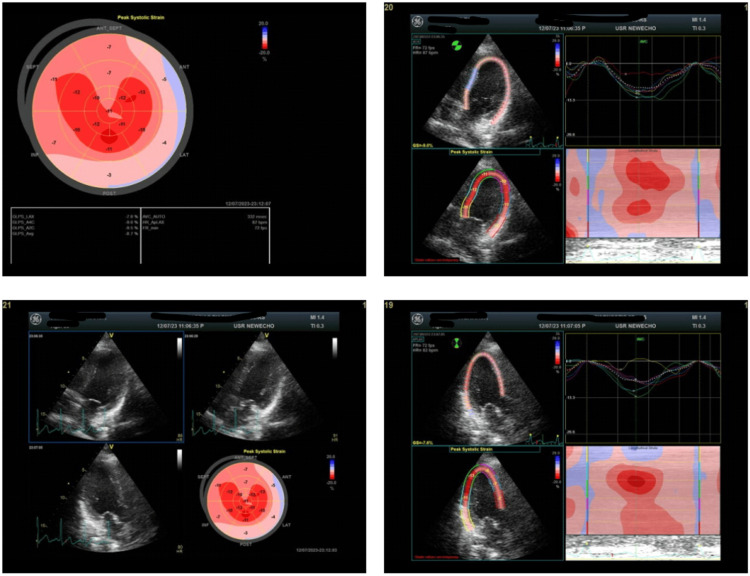
Case 2: Bull's eye image of left ventricular global longitudinal strain imaging Bull's eye image of left ventricular global longitudinal strain imaging showing a moderate reduction in average global longitudinal strain values.

A 35-year-old primigravida presented with class III dyspnea at 36 weeks of gestation. She had a history of conception one year after marriage and had undergone an LSCS at 37+2 weeks. Her ECG showed normal sinus rhythm. Two-dimensional echocardiography showed global LV hypokinesia with LVEF of 40% and LV GLS of -8.7% (Figure 2), indicative of moderate impairment of cardiac function. She was euthyroid with a history of gestational diabetes (managed with diet, lifestyle modification, and metformin). She also had pregnancy-induced hypertension (controlled on nifedipine and labetalol). She received ramipril, carvedilol, furosemide, and bromocriptine. Her response to treatment was closely monitored, with a focus on alleviating dyspnea and improving cardiac function. Regular assessments of LV GLS were performed to quantify myocardial response to therapy. Over subsequent weeks, the patient showed improvement in dyspnea and overall clinical status. LV GLS was -11.2%, with LVEF of 45%, indicating improvement in myocardial function.

Case 3

**Figure 3:**
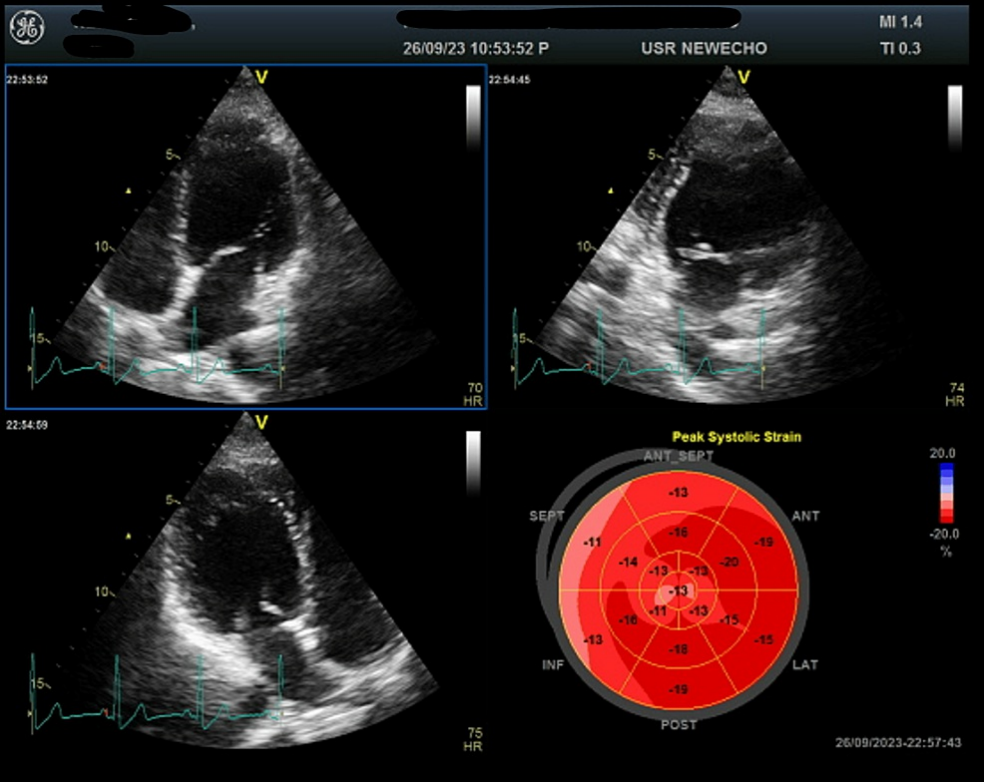
Case 3: Bull's eye image of left ventricular global longitudinal strain imaging Bull's eye image of left ventricular global longitudinal strain (LV GLS) imaging indicative of mild impairment of cardiac function with a mild reduction in average LV GLS values.

A 30-year-old primigravida who conceived through in vitro fertilization presented with class III dyspnea on postpartum day two after a cesarean section at 38+1 weeks. ECG showed normal sinus rhythm with global LV hypokinesia and LVEF of 45% on echocardiography and LV GLS of -14.2% (Figure 3), indicative of mild impairment of cardiac function with a reduction in LV GLS. She had a history of pregnancy-induced hypertension but no gestational diabetes mellitus and was euthyroid. She received ramipril, bisoprolol, and eplerenone. Over the postpartum period, the patient showed improvement in dyspnea, and her LV GLS values demonstrated a significant improvement (-16.9%, with LVEF of 55%), indicating recovery of myocardial function.

## Discussion

Global longitudinal strain (GLS) stands out with broad applicability in various LV heart diseases. It exhibits enhanced reliability compared to LVEF, especially in the assessment of sequential measures. Notably, it demonstrates high precision and superior reproducibility when contrasted with conventional measures like E, A, e', and LVEF. Furthermore, it contributes additional prognostic value to the evaluation. The onset of heightened strain serves as an initial indicator of cardiac impairment preceding the onset of clinical heart failure. This has proven especially valuable in assessing the cardiotoxic effects of chemotherapy. The potential extension of its utility to various subclinical cardiomyopathies is a topic of significant interest, considering the convenience and cost-effectiveness associated with seamlessly incorporating strain measures into standard echocardiographic imaging protocols [4].

Prior research has emphasized the diagnostic utility of LV GLS in identifying subclinical myocardial dysfunction within the obstetrical context, including conditions like pre-eclampsia and gestational diabetes mellitus. The results of this study reveal that term preeclamptic pregnant women, despite showing minimal functional alterations on conventional echocardiography, exhibit noteworthy subclinical myocardial changes when assessed through speckle tracking analysis [5,6].

In an independent study conducted at a single center involving 68 women, both GLS and LVEF experienced a notable decrease during the late stages of pregnancy. However, they rebounded to baseline levels after delivery. This introduces a point of contention regarding the alterations in GLS, even within the context of a healthy pregnancy. The variation in strain could potentially differ based on factors such as trimester, whether it is a single or multiple gestation, maternal age, and the presence of other pregnancy-related comorbidities, such as diabetes, which has been associated with reduced GLS. The identification of women at risk and the ability to predict the prognosis of established PPCM could prove valuable in the effective management of the disease [7].

We share our single-center experience involving three cases of PPCM, accompanied by their short-term follow-up. Our study indicates that incorporating changes in LVEF and LV GLS provides additional insight into the prognosis of PPCM. We aim to assess the utility of LV GLS in the routine evaluation of antenatal care cases to determine its predictive value for the development of PPCM in the later stages of pregnancy. Additionally, we explore the potential of serial assessments of LV GLS in predicting the recovery of LVEF in confirmed PPCM cases. Furthermore, our investigation extends to understanding the outcomes and long-term prognosis of PPCM cases, particularly those with recovered LVEF but persistent abnormalities in LV GLS values.

LV GLS offers a sensitive and early indicator of myocardial dysfunction, enabling the timely identification of subtle changes in cardiac performance that may precede overt symptoms. Monitoring LV GLS during the treatment and recovery phases provides valuable insights into the efficacy of prescribed therapies, facilitating timely adjustments to optimize patient outcomes. To consider GLS as a tool for present and future therapeutic advancement, further research involving more diverse cohorts and varied settings is warranted to enhance the external validity of our findings. It will also provide a more comprehensive understanding of the broader implications of the routine use of LV GLS in the diagnosis and management of PPCM [4].

## Conclusions

These cases highlight the importance of LV GLS imaging in the context of PPCM by providing valuable quantitative data, thus aiding in the assessment of myocardial function and guiding therapeutic decisions. The positive response observed in all cases underscores the significance of a comprehensive approach to management, considering both clinical and imaging parameters.
